# DNA identification of species of the *Anopheles maculipennis* complex and first record of *An. daciae* in Belgium

**DOI:** 10.1111/mve.12519

**Published:** 2021-05-05

**Authors:** N. Smitz, K. De Wolf, A. Gheysen, I. Deblauwe, A. Vanslembrouck, K. Meganck, J. De Witte, A. Schneider, I. Verlé, W. Dekoninck, S. Gombeer, A. Vanderheyden, M. De Meyer, T. Backeljau, R. Müller, W. Van Bortel

**Affiliations:** ^1^ Royal Museum for Central Africa (BopCo & Biology Department) Tervuren Belgium; ^2^ The Unit of Entomology, Department of Biomedical Sciences Institute of Tropical Medicine Antwerp Belgium; ^3^ Royal Belgian Institute of Natural Sciences (BopCo & Scientific Heritage Service) Brussels Belgium; ^4^ Evolutionary Ecology Group University of Antwerp Antwerp Belgium; ^5^ Outbreak Research Team Institute of Tropical Medicine Antwerp Belgium

**Keywords:** *Anopheles maculipennis* subgroup, cytochrome oxidase I (COI), internal transcribed spacer 2 (ITS2), malaria vector, occurrence, restriction fragment length polymorphism (RFLP)

## Abstract

The present study aimed at identifying the members of the *Anopheles maculipennis* complex (Diptera: Culicidae) occurring in Belgium. Therefore, the second internal transcribed spacer of nuclear ribosomal DNA (ITS2) and the mitochondrial cytochrome oxidase subunit I (COI) loci were sequenced in 175 and 111 specimens, respectively, collected between 2007 and 2019. In parallel, the suitability of two species‐diagnostic PCR‐RFLP assays was tested. The identified specimens included: *An. maculipennis* s.s. (N = 105), *An. daciae* (N = 62), *An. atroparvus* (N = 6) and *An. messeae* (N = 2). Each species was characterized by unique ITS2 haplotypes, whereas COI only supported the monophyly of *An. atroparvus*, a historical malaria vector in Belgium. Species identification results were further supported by unique PCR‐RFLP banding patterns. We report for the first time *An. daciae* in Belgium, where it was found to co‐occur with *An. maculipennis* s.s. The latter was the most prevalent in the collection studied (60%) and appears to have the widest distribution in Belgium*.* As in other studies, *An. daciae* and *An. messeae* appeared the most closely related species, up to the point that their species status remains debatable, while their ecological differences, including vector competences, need further study.

## Graphical abstract

Species identification of 175 specimens of *Anopheles maculipennis* s.l. collected in Belgium, comparing the performance of COI and ITS2 loci, and two species‐diagnostic PCR‐RFLP assays.
Four species identified: *An. maculipennis* s.s. (N = 105), *An. daciae* (N = 62), *An. atroparvus* (N = 6) and *An. messeae* (N = 2), with *An. daciae* reported for the first time in Belgium.
Each species was characterized by unique ITS2 haplotypes, whereas COI only supported the monophyly of *An. atroparvus* (malaria vector). *Anopheles daciae* and *An. messeae* appeared the most closely related.

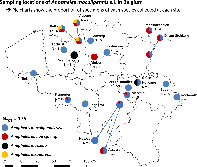

## Introduction

In the Paleartic region, ten cryptic species of the *Anopheles maculipennis* subgroup (Maculipennis complex) are presently recognized (Harbach, 2013), with *An. messeae* Falleroni, 1926 being the most widely distributed (Becker *et al*., 2020). Seven of these cryptic species are distributed throughout continental Europe, viz. *An. atroparvus* van Thiel, 1927, *An. daciae* Linton, Nicolescu & Harbach, 2004, *An. labranchiae* Falleroni, 1926, *An. maculipennis* s.s. Meigen, 1818, *An. melanoon* Hackett, 1934, *An. messeae* and *An. sacharovi* Favre, 1903.

Because several pathogens were isolated from species of the Maculipennis complex, including malaria (Filipe, 1972; Lindsay & Birley, 1996; Cancrini *et al*., 1997, 2006; Jöst *et al*., 2010, 2011), it is important to be able to identify them accurately and to map their distribution for monitoring programs. Furthermore, some closely related Maculipennis species display very different ecological, behavioural and physiological characteristics that may affect their vector status (Jetten & Takken, 1994; Takken & Verhulst, 2013). For example, while in 2015 the WHO European region was declared free of indigenous malaria, there has been a substantial increase of imported tropical malaria over the last two decades, resulting from travel and mass immigration. Given the presence of competent *Anopheles* vectors of malaria in Europe, the import of tropical malaria has led to the reappearance of autochthonous malaria cases in France, Italy, Greece and Cyprus (European Centre for Disease Prevention and Control, 2017). Also, environmental impacts due to climate change may contribute to changes in species distribution and expanding vector ranges (Lindsay & Birley, 1996). Therefore, it is important to monitor the distribution of *Anopheles* species across Europe and investigate their link with human travel and climate change. This can only be achieved if Maculipennis species are correctly identified.

In Belgium, three species of the Maculipennis complex have been reported: *An. atroparvus*, *An. messeae* and *An. maculipennis* s.s. (Boukraa *et al*., 2015). *Anopheles atroparvus* has not been collected in Belgium during the nationwide inventory between 2007 and 2010 (Versteirt *et al*., 2013). This may probably reflect the decline over the last century of the species, as observed in other European countries (van Seventer, 1970; Takken *et al*., 2002). Within the Maculipennis complex, species are morphologically indistinguishable when larvae, pupae or adults are considered. The egg morphology has traditionally been used to identify the species (Korvenkontio *et al*., 1979; WHO, 2008). Unfortunately, this is not foolproof, since egg characteristics show overlapping intraspecific, geographic and seasonal variations among species (Sedaghat *et al*., 2003), with for example *An. atroparvus* and *An. messeae* being hardly distinguishable based on egg morphology (Rodhain & van Hoof, 1942). *Anopheles daciae* is the most recent described species of the complex (Nicolescu *et al*., 2004) and it co‐occurs with *An. messeae* in Czech Republic (Blažejová *et al*., 2018), England (Danabalan *et al*., 2014), Finland (Culverwell *et al*., 2020), Germany (Weitzel *et al*., 2012), Greece (Linton *et al*., 2001), Italy (Di Luca *et al*., 2004), Poland (Rydzanicz *et al*., 2017), Romania (Nicolescu *et al*., 2004), Serbia (Kavran *et al*., 2018), Slovakia (Blažejová *et al*., 2018), Sweden (Lilja *et al*., 2020) and Wales (Danabalan *et al*., 2014).

Since morphological characteristics are not reliable for the identification of the Maculipennis species, DNA approaches have been explored as an alternative tool. The nuclear ribosomal internal transcribed spacer 2 (ITS2) flanked by portions of the conserved 5.8S and 28S rDNA where the primers anneal is useful in this respect (Proft *et al*., 1999;Nicolescu *et al*., 2004; Danabalan *et al*., 2014). This DNA fragment usually displays high degree of interspecific differentiation, but low intraspecific variation, which makes it suitable for the identification of closely related *Anopheles* species (Collins & Paskewitz, 1996). Still, between *An. daciae* and *An. messeae*, ITS2 shows only five putatively species‐specific single nucleotide polymorphisms (SNPs) (Nicolescu *et al*., 2004), or two if most recent studies are considered (Culverwell *et al*., 2020; Lilja *et al*., 2020).

Next to ITS2 sequencing, some Polymerase Chain Reaction‐Restriction Fragment Length Polymorphism (PCR‐RFLP) assays were proposed to identify species of the Maculipennis complex, as a cheaper and faster identification method for monitoring activities. Two of these assays were considered suitable by producing sufficiently different sized fragments after restriction of the ITS2 gene (Nicolescu *et al*., 2004; Danabalan *et al*., 2014). Also, the mitochondrial Cytochrome c oxidase subunit 1 (COI) DNA fragment, which is recognized as a powerful tool for the identification of many culicid taxa, was proposed to be able to separate *An. daciae* with its unique mitochondrial COI DNA sequences (Nicolescu *et al*., 2004; Linton *et al*., 2005). However, the available COI database of the latter species in online repositories is presently limited to sequences from Romania, while COI was also found to weakly support *An. maculipennis* s.s. and *An. messeae* in Belgium (Versteirt *et al*., 2015). Therefore, the present study explores and applies ITS2 sequencing to identify the members of the Maculipennis complex in Belgium (standard species identification technique of the complex members) and compares its suitability as identification tool for this species complex with COI sequencing and two PCR‐RFLP assays. This work provides the first solid evidence on the occurrence of *An. daciae* in Belgium.

## Methods

### Sampling

*Anopheles maculipennis* s.l. larvae and adults were collected in the framework of different successive projects undertaken to evaluate the Belgian mosquito biodiversity and distribution, and to monitor the introduction and establishment of exotic mosquito species in Belgium (Versteirt *et al*., 2013; Deblauwe *et al*., 2015, 2020). A total of 175 specimens were selected from the collections between 2007 and 2019 from 25 locations (Table S1). Larvae were collected using aquarium nets in different types of breeding sites, but with a focus on those of invasive *Aedes* species. Breeding sites included ditches, gutters, road drains, catch basins, artificial containers (metal, plastic, glass, stone), tyres, plastic sheets, ponds, puddles and tree holes. Sampling strategies and methodologies are detailed in Versteirt *et al*. (2013) and Deblauwe *et al*. (2015, 2020). Larvae and adults were morphologically identified as *An. maculipennis* s.l. following Gunay *et al*. (2018) and Becker *et al*. (2020), and subsequently preserved in 80% ethanol (larvae) or dry (adults) at room temperature for DNA analyses. Specimens and dried DNA extracts are stored in the collections of the Royal Belgian Institute of Natural Sciences (RBINS:IG32776; RBINS:IG34179).

To further characterize the habitat of *Anopheles maculipennis* s.l., the Corine Land Cover Classes were calculated in a 2.5 km buffer zone around each location using the latest raster file (Copernicus, 2021) in Q‐GIS, with calculations made with RStudio (RStudioTeam, 2020). The levels were grouped into five classes (*i.e*. artificial or urban areas, agricultural areas, forest and seminatural areas, wetlands and water bodies).

### DNA species identification

#### DNA extraction, PCR amplification and sequencing

Individual DNA was extracted from legs, parts of abdomens, or complete specimens (in case of first larval stages) using either the NucleoSpin® Tissue DNA extraction kit (Macherey‐Nagel, Düren, Germany) or the QIAamp DNA Micro kit (Qiagen, Venlo, Netherlands), following the manufacturer's protocols, except that the elution volume was set to 70 μL. The ITS2 and COI gene fragments were amplified using the primers and PCR cycling conditions described in Weitzel *et al*. (2012) and van de Vossenberg *et al*. (2013). The thermal cycling conditions for the amplification of ITS2 were adapted as follows: 3 min denaturation at 94 °C, 35 cycles at 94 °C for 30 s, 46 °C for 30 s and 72 °C for 45 s, followed by a final 7 min elongation at 72 °C. PCR reactions, purification and sequencing of both strands were carried out as described in Ibáñez‐Justicia *et al*. (2020). The quality of the sequencing output was checked with Geneious® Prime (Biomatters Ltd., Auckland, New Zealand) software, after which sequences were trimmed, corrected and assembled.

Generated consensus sequences and outgroup sequences (*An. plumbeus* Stephens, 1828, *An. claviger* (Meigen, 1804), *An. algeriensis* Theobald, 1903) were aligned using ClustalW in Geneious® Prime (outgroup COI GenBank accession numbers: KM258216, MK402896, MK402867; outgroup ITS2 GenBank accession numbers: MK412752, DQ229313, MK412758). The online application FindModel was used to check which evolution model best describes our data (Posada & Crandall, 1998; Tao *et al*., 2016), namely the Kimura 2‐parameter and the Tamura‐Nei models for ITS2 and COI, respectively. Rooted haplotype maximum likelihood trees (ML) were constructed using MEGA X (Kumar *et al*., 2018), with branch support assessed by 1000 bootstrap replicates and pairwise deletion of indels. Condensed trees with cut‐off value of 50% are presented, and COI and ITS2 haplotype alignments are provided as supporting information (Appendices S1 and S2).

Haplotypes were then used as queries to search for most similar sequences in the public online database GenBank (NCBI, National Centre for Biotechnology) for species identification, using the Basic Local Alignment Search Tool (https://blast.ncbi.nlm.nih.gov/Blast.cgi). To discriminate between *An. daciae* and *An. messeae*, however, aligned consensus sequences were visually checked for the presence of the five species‐specific diagnostic sites (Nicolescu *et al*., 2004).

Once specimens were identified by ITS2 sequencing, average interspecific K2P distances and maximum observed K2P distances between conspecific COI sequences, were calculated with the package Spider v3.6.2 (Brown *et al*., 2012; RStudioTeam, 2020). Pairwise differences in nucleotide frequencies between species were evaluated using Wright's *F*‐statistics, as implemented in Arlequin v3.5 (1000 random permutations for significance, with subsequent standard Bonferroni correction). Haplotype frequencies, mean numbers of pairwise nucleotide differences (*k*) and average gene diversities over nucleotide positions (*H*) were also calculated with Arlequin.

#### RFLP assays

To test the usefulness of the RFLP assays for species identification, ITS2 PCR products were further processed with two restriction enzymes that produce species‐diagnostic banding patterns for the Maculipennis complex, viz. Hha I (CGC↓G) to distinguish between *An. maculipennis*, *An. atroparvus* and *An. daciae/An. messeae* (Nicolescu *et al*., 2004), and Bsh 1236I (CG↓CG) to differentiate *An. daciae*, *An. messeae* and *An. atroparvus* (Danabalan *et al*., 2014). Half of the purified ITS2 PCR product was digested using FastDigest Hha I (Thermo Fisher Scientific, Waltham, MA, USA), the other half was digested using FastDigest Bsh 1236I (Thermo Fisher Scientific). For both reactions, the total reaction volume was 30 μL, comprising 17 μL nuclease‐free water, 2 μL 10× FastDigest Green Buffer, 1 μL FastDigest enzyme and 10 μL of purified PCR product. Samples were incubated at 37 °C in a ThermoMixer for 7 min. Restriction fragments were size‐separated by electrophoresis on a 3% agarose gel (1 h at 80 V), together with the FastGene 50 bp DNA ladder (NIPPON Genetics Europe, Düren, Germany). Visualization was performed on a UV transilluminator using the MidoriGreen™ Direct (NIPPON Genetics Europe) staining method.

## Results

The ITS2 fragment was scored in 175 specimens and sequences were deposited in GenBank (accession numbers: *An. atroparvus*: MT514842‐MT514847; *An. daciae*: MT514848‐MT514909; *An. maculipennis* s.s.: MT514737‐MT514841; *An. messeae*: MT514735‐MT514736). Amplicon sizes varied from 472 bp in *An. maculipennis* s.s., 485 bp in both *An. daciae* and *An. messeae*, to 487 bp in *An. atroparvus*. Hence, *An. daciae* and *An. messeae* could not be identified by PCR fragment gel electrophoresis.

**Fig. 1:**
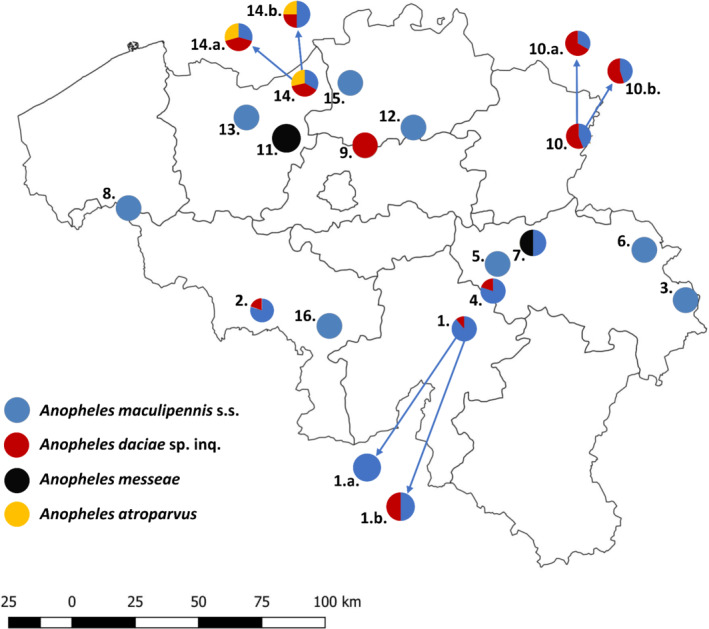
Sampling locations of *Anopheles maculipennis* s.l. in Belgium. The pie charts show the proportion of specimens of each species collected at each location. Locality identifier and the number of specimens analysed per site are presented in Table S1.

ITS2 sequences allowed to assign six specimens to *An. atroparvus*, two to *An. messeae*, 62 to *An. daciae* and 105 to *An. maculipennis* s.s. (Table S1). *Anopheles atroparvus* specimens were all collected at the locations Kallo (N = 5) and Vrasene (N = 1) (Fig. 1) using the Mosquito Magnet and the BG‐Sentinel traps over two successive years (three specimens collected in June, July and August 2018; three in July and August 2019). *Anopheles messeae* was collected twice with the Mosquito Magnet trap, in 2007 and 2013 at Grembergen and Grâce‐Hollogne, respectively, both locations displaying a high percentage of agricultural areas (Tables S1 and S2). *Anopheles maculipennis* s.s. was the most common (60%) and widespread species, occurring in sympatry with *An. daciae* at eight locations (Fig. 1 and Table S1). Larvae and adults of *An. daciae* were recorded at 11 coordinates (Table S1). The latter species is reported for the first time in Belgium. Adults of both *An. maculipennis* s.s. and *An. daciae* species were collected with the Mosquito Magnet and the BG‐Sentinel traps. Larval breeding sites of *An. maculipennis* s.s. were mainly artificial containers (metal, plastic, stone), a plastic sheet, tyres, road drains and ditches, while those of *An. daciae* were a pond and a metal container (bath tub used as animal drinking trough).The highest numbers of both *An. maculipennis* s.s. and *An. daciae* were collected at locations dominated by forest and seminatural areas (Tables S1 and S2).

None of the ITS2 haplotypes was shared between any of the four species of the Maculipennis complex in Belgium, *i.e*. each of the four species involved one single, species‐specific haplotype (Fig. S1). The number of ITS2 diagnostic sites between species varied from 36 (including 2 gaps) for *An. atroparvus*, 21 (including 12 gaps) for *An. maculipennis* s.s., to two for both *An. daciae* and *An. messeae*. The average interspecific K2P distances ranged from 0.457 to 7.702% (Table 1). However, three of the five supposedly diagnostic sites discriminating *An. messeae* from *An. daciae* showed sometimes double peaks in *An. daciae* (N_TOT_ = 62), viz. position 214 (A/T) (N = 2), 218 (A/T) (N = 10) and 221 (C/T) (N = 2) (site numbering following Nicolescu *et al*., 2004), which represents 6.2% of the generated *An. daciae* sequences. In contrast, the two Belgian ITS2 sequences of *An. messeae* showed no ambiguities at the five discriminative sites (214 (T), 218 (T), 221 (C), 416 (G), 436 (G)).

**Table 1:** Descriptive statistics of the genetic diversity of COI and ITS2 within *Anopheles maculipennis* s.l. in Belgium, including the overall maximum observed intraspecific Kimura two‐parameter (K2P) distances among COI sequences, and average interspecific K2P distances among COI and ITS2 sequences.

	COI							ITS2			
	N	N_H_	N_P_	*k* ± SD	*H* ± SD	Average interspecific K2P (%)	Max intraspecific K2P (%)	N	N_H_	N_P_	Average interspecific K2P (%)
*An. atroparvus*	6	6	13	5.467 ± 3.023	0.009 ± 0.006	2.405	1.271	6	1	0	7.702
*An. daciae* sp. inq.	49	39	44	6.684 ± 3.203	0.011 ± 0.006	0.816	2.786	62	1	0*	1.119
*An. maculipennis* s.s.	54	39	34	7.462 ± 3.536	0.013 ± 0.007	2.273	2.644	105	1	0	3.170
*An. messeae*	2	2	13	13.00 ± 9.210	0.029 ± 0.028	0.186	2.942	2	1	0	0.457

N, sample size; N_H_, number of haplotypes; N_P_, number of polymorphic loci; *k*, mean number of pairwise nucleotide differences, *H*, average gene diversity over nucleotide positions; SD, standard deviation.

^*^ Ambiguities recorded at three of the five species‐diagnostic sites in 6.2% of the *An. daciae* specimens.

The COI fragment was scored in 111 specimens (Table 1), and sequences were deposited on GenBank (accession numbers MT769652‐MT769762). As expected, COI displayed more intraspecific variation than ITS2, with two to 39 distinct haplotypes recognized within each species (Table 1). Base changes were all silent, mainly occurring at the third codon position. *Anopheles atroparvus* was the only species having diagnostic sites (N = 9) with the other members of the complex. COI ML‐tree only supported *An. atroparvus* haplotypes as a distinct cluster (Fig. S2). Maximum intraspecific K2P distances among COI sequences varied from 1.271 to 2.942%, while the average interspecific K2P distances ranged from 0.186 to 2.786% (Table 1). The lowest interspecific distance was obtained between *An. daciae* and *An. messeae*, accompanied by a low and non‐significant COI *F*_*ST*_ value (Table S3).

**Fig. 2:**
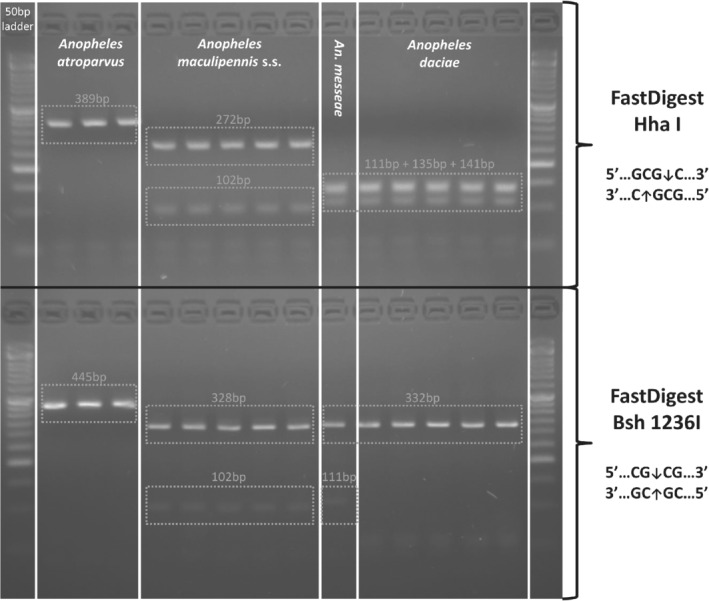
RFLP patterns of *Anopheles maculipennis* s.l. from Belgium obtained by electrophoresis in 3% agarose gels of Hha I and Bsh 1236I enzyme digestions of ITS2 amplicons.

Finally, ITS2 species‐diagnostic RFLP patterns were revealed on a 3% agarose gel (Fig. 2), with all expected bands being visible. For Hha I, *An. maculipennis* s.s. (42, 56, 102, 272 bp) and *An. atroparvus* (42, 56, 389 bp) can clearly be discriminated from *An. daciae/An. messeae* (42, 56, 111, 135, 141 bp). For Bsh 1236I, *An. daciae* (42, 52, 59, 332 bp), *An. messeae* (42, 111, 332 bp) and *An. atroparvus* (42, 445 bp) have a unique banding pattern, but *An. messeae* is hardly distinguishable from *An. maculipennis* s.s. (42, 102, 328 bp) due to small fragment size differences *i.e*. 111 bp *vs* 102 bp and 332 bp *vs* 328 bp (Fig. 2).

## Discussion

Four species of the Maculipennis complex were identified in Belgium, viz. *An. maculipennis* s.s., *An. daciae*, *An. messeae* and *An. atroparvus*. The present result is the first report of *An. daciae* in Belgium. Species identification by ITS2 sequences and RFLP patterns of Hha I and Bsh 1236I were consistent, confirming earlier studies (Nicolescu *et al*., 2004; Danabalan *et al*., 2014). Hence, the RFLP method represents a cost‐efficient and rapid alternative for the routine identification of the four *An. maculipennis* s.l. species in Belgium, provided that both restriction enzymes (Hha I and Bsh 1236I) are used jointly. This is important, since RFLP patterns of Hha I do not differentiate *An. daciae* from *An. messeae*, while RFLP patterns of Bsh 1236I hardly differentiate *An. maculipennis* s.s. from *An. messeae*. ITS2 was fixed for a single haplotype in *An. atroparvus*, *An. messeae* and *An. maculipennis* s.s. Ambiguous sites at three of the five species‐diagnostic sites were observed in ten *An. daciae* ITS2 sequences (position 214 (A/T), 218 (A/T) and 221 (C/T)). Double peaks can result from slight differences among ITS2 copies within and/or between chromosomes (heterozygosity) (Collins & Paskewitz, 1996). This phenomenon was previously reported in *An. daciae* by Bezzhonova & Goryacheva (2008), Culverwell *et al*. (2020) and Lilja *et al*. (2020). The two other species‐specific sites discriminating *An. daciae* from *An. messeae* were located at positions 416 (A/G) and 436 (C/G) (Nicolescu *et al*., 2004). Therefore, the latter two positions might actually be the only diagnostic sites allowing to differentiate both species. Additionally, the limited intraspecific ITS2 variation within all species of the complex may be underestimated because of poor geographic sampling in most studies published up to now. A survey of ITS2 variation in *An. messeae* populations in Russia reported nine ITS2 variants (Bezzhonova & Goryacheva, 2008). When investigating *An. messeae* and *An. daciae* ITS2 sequences from specimens collected at a larger geographical scale (England, Wales, China, Germany, Italy, Russia, Kazakhstan and former Yugoslavia), only one single ITS2 diagnostic site seems to remain (Danabalan *et al*., 2014), namely C/G at position 436.

The variation at the COI DNA fragment makes that this marker does not look promising to discriminate the members of the Maculipennis complex. Similarly, ND5, ND4 and the Hunchback gene fragments seem ineffective to discriminate *An. daciae* from *An. messeae*, since the haplotypes of both species are not clustering in phylogenetic trees based on these gene fragments (Lilja *et al*., 2020). All DNA sequence data support a close relationship between *An. daciae* and *An. messeae*, which is in line with all previous studies investigating these two taxa. Currently, ITS2 might be the only useful species marker, since, with its rapid evolutionary turnover within and between rDNA repeats, it has a higher ability to manifest early genetic discontinuities than other genes (Collins & Paskewitz, 1996), resulting in higher inter‐ and lower intraspecific genetic distances within the Maculipennis complex.

Given the debatable DNA evidence of the specific distinction between *An. daciae* and *An. messeae*, it is important to look for other possible evidence that can be relevant for the taxonomic interpretation of these two nominal species. One such argument may be the suggestion that *An. daciae* may also feed on humans (Danabalan *et al*., 2014), whereas *An. messeae* appears strictly zoophilic (Danabalan *et al*., 2014; Brugman *et al*., 2015). However, these observations are preliminary based on a single study, involving a small sampling size from a limited geographic area, while feeding preferences may be driven by the availability of hosts (Chaves *et al*., 2010). In general, taxonomic discriminative features between *An. daciae* and *An. messeae* (e.g. hybrid incompatibility, morphology, ecology, cytotaxonomy, zymotaxonomy, vector competencies, etc.) are still poorly known. Further investigations on these aspects could, additionally validate the taxonomic status of these nominal species, help understanding the potential role of *An. daciae* in the historical transmission of malaria in the Palearctic region. Until then, *An. daciae* should be referred to as *species inquirenda*, i.e. a species of doubtful identity as defined by the International Commission on Zoological Nomenclature (ICZN, 1999).

The occurrence of a particular species of the Maculipennis complex can vary largely across their overlapping distribution ranges. In Germany, *An. messeae* is the predominant species of the complex (Lühken *et al*., 2016), whereas in England *An. daciae* sp. inq. is predominant (Danabalan *et al*., 2014), with both species found to co‐occur in a variety of breeding sites (Kavran *et al*., 2018). Only *An. atroparvus*, a predominant species in brackish water, is consistently found in low densities in West and Central Europe (Weitzel *et al*., 2012; Lühken *et al*., 2016; Kavran *et al*., 2018). This species was the main vector of malaria in Western Europe and in Belgium (Rodhain & van Hoof, 1942; Mouchet *et al*., 2004). In Belgium, the species occurred along the coast, near Antwerp up to Limburg, with one observation in Namur, and was primarily linked to brackish water (Rodhain & van Hoof, 1942). In the current study, we found the species in an area where it was historically present. The current distribution of *An. atroparvus* in Belgium is unknown. Rodhain & van Hoof (1942) already speculated that the important drainage of wetlands in Flanders, which started in the mid‐19th century, contributed to the decline of this species. However, brackish environments have become very scarce in Belgium (Perillo *et al*., 2009). Furthermore, the species' significant decline in Europe over the last century was also proposed to be linked to surface water pollution, the loss of suitable resting sites for hibernation, the competition with more ubiquitous species and the application of insecticides (Rodhain & van Hoof, 1942; van Seventer, 1970; Takken *et al*., 2002).

From the present study, *An. maculipennis* s.s. appears by far the most common and widespread species of the complex in Belgium, confirming results from Versteirt *et al*. (2013). However, a sampling bias towards the collection of *An maculipennis* s.s. specimens can occur, since the main goal of the monitoring programs undertaken in Belgium was to intercept exotic *Aedes* mosquitoes, which often coincides with locations predominantly composed of man‐made breeding sites. The latter are usually characterized by higher quantities of ammonia and mud (eutrophic water), to which *An. maculipennis* s.s. seems to be better adapted (Weyer, 1938; Dakić *et al*., 2008; Becker *et al*., 2020). *Anopheles messeae*/*An. daciae* sp. inq. are more selective and frequently found in ponds or larger artificial containers filled up with cleaner water, either stagnant or slow moving, as inundation areas of rivers and lake systems (Linton *et al*., 2002; Dakić *et al*., 2008; Weitzel *et al*., 2012). From the present results, it seems that *An. daciae* sp. inq. was captured at places located nearby nature reserve comprising ponds and wetlands (Table S1 – e.g. Kallo: Verrebroekse blikken, Dilsen‐Stokkem: National Park Hoge Kempen closeby Terhill, Muizen: Mechels Broek) with highest numbers of collected adults in forest and seminatural areas. The present findings provide the current known occurrence of the Maculipennis species in Belgium, though it may not reflect a precise distribution of the species as the observations are based on surveillance primarily aiming to detect exotic mosquito species. In the future, developing a targeted nationwide surveillance program would be of value given the role of some Maculipennis species in disease transmission.

## Author contributions

Conceptualization and design of the study: NS, KDW, ID, WD, MDM, TB, WVB; Acquisition of data: NS, KDW, AG, ID, AV, KM, JDW, AS, IV, SG, AV; Analysis and interpretation of data: NS, KDW, AG, ID, KM, SG, AV, MDM, TB, RM, WVB; drafting manuscript and revising: NS, KDW, AG, ID, AV, KM, JDW, AS, IV, WD, SG, AV, MDM, TB, RM, WVB. All authors have read and agreed to the published version of the manuscript.

## Supporting information

**Appendix S1.** Generated COI haplotypes aligned using ClustalW in Geneious® Prime.

**Appendix S2.** Generated ITS2 haplotypes aligned using ClustalW in Geneious® Prime.

**Fig. S1.** Condensed ITS2 haplotype ML‐tree of four members of *Anopheles maculipennis* s.l. in Belgium (Kimura 2‐parameter model), with *An. plumbeus*, *An. claviger* and *An. algeriensis* as outgroup. Numbers at nodes are bootstrap support values >50%.

**Fig. S2.** Condensed COI haplotype ML‐tree of four members of *Anopheles maculipennis* s.l. in Belgium (Tamura‐Nei model), with *An. plumbeus*, *An. claviger* and *An. algeriensis* as outgroup. Numbers at nodes are bootstrap support values >50%.

**Table S1.** Sampling locations (Fig. 1), Corine Land Cover Class (class with highest percentage in a 2.5 km buffer zone around the location (group levels based on five classes, Table S2)) and DNA‐based identification results of specimens collected in Belgium from 2007 until 2019 with indication of the life stage at collection (A, number of adult specimens; L, number of larvae), the adult trap type which collected the specimens and the type of sampled breeding site positive to *Anopheles maculipennis* s.l. larvae. When multiple coordinates are reported for one location in the table, the middle point between the coordinates was used to position the pie chart on Fig. 1. MMT, mosquito magnet trap; BG, BG‐sentinel trap; GT, gravid trap.

**Table S2.** Corine Land Cover Classes in a 2.5 km buffer zone around the locations with *Anopheles maculipennis* s.l. collections, with indication of the highest percentage (bold).

**Table S3.** Pairwise *F*_*ST*_ estimates between species of the *An. maculipennis* complex based on COI, calculated using Arlequin v3.5. Significant values after standard Bonferroni correction marked by an asterisk (*P* < 0.0005).

## Data Availability

The data that support the findings of this study are openly available in GenBank at https://www.ncbi.nlm.nih.gov/genbank/, accession numbers: MT514735‐MT514909, MT514735‐MT514841, and MT769652‐MT769762.
