# Supplementary figures and images for: DNA identification of species of the Anopheles maculipennis complex and first record of An. daciae in Belgium
Source: Med Vet Entomol. 2021 May 5;35(3):442–50. doi: 10.1111/mve.12519 (PMC8453948; doi:10.1111/mve.12519)

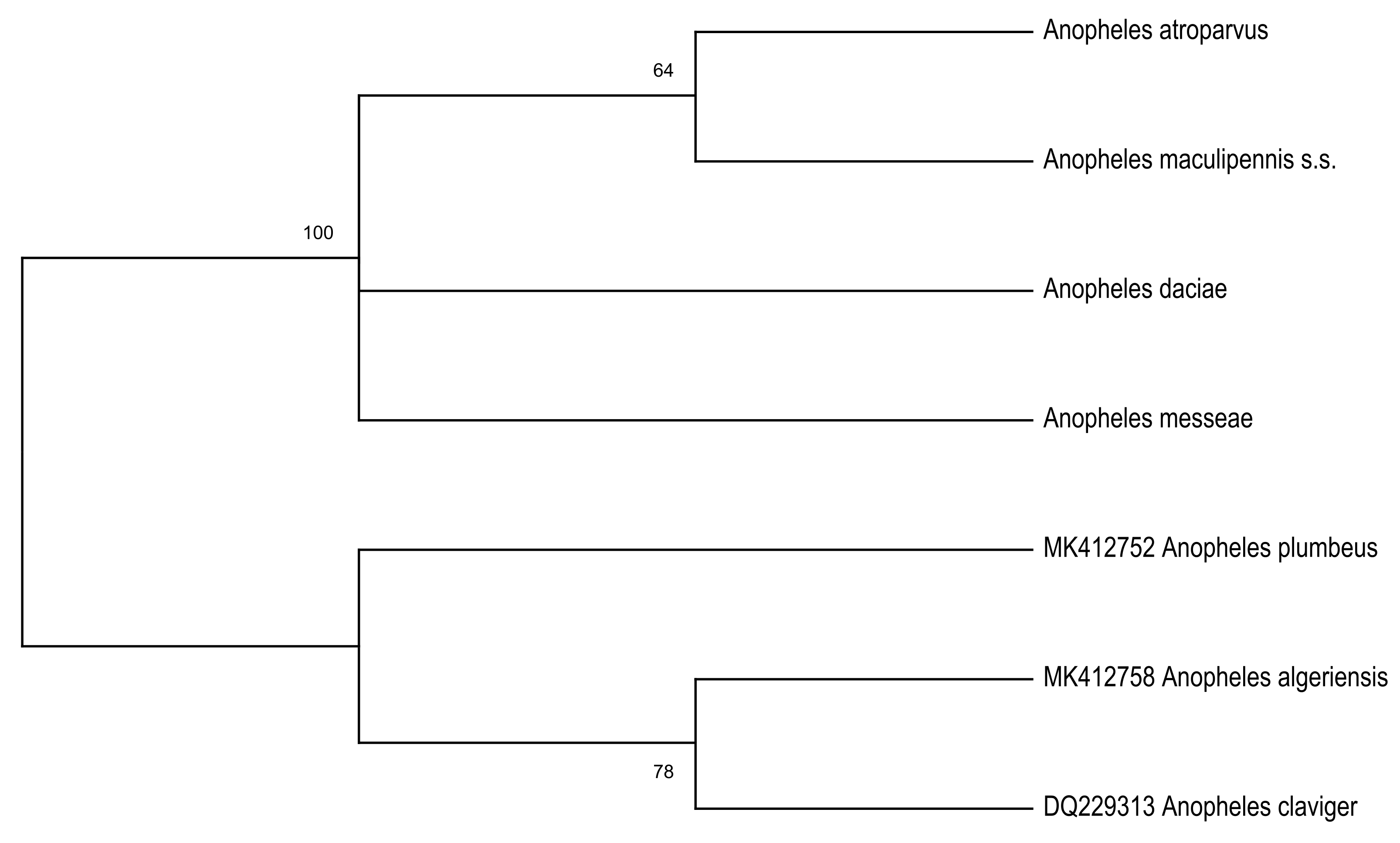

Supplement: Supplementary file 3 — Fig. S1. Condensed ITS2 haplotype ML‐tree of four members of Anopheles maculipennis s.l. in Belgium (Kimura 2‐parameter model), with An. plumbeus, An. claviger and An. algeriensis as outgroup. Numbers at nodes are bootstrap support values >50%. [file MVE-35-442-s007.tif]

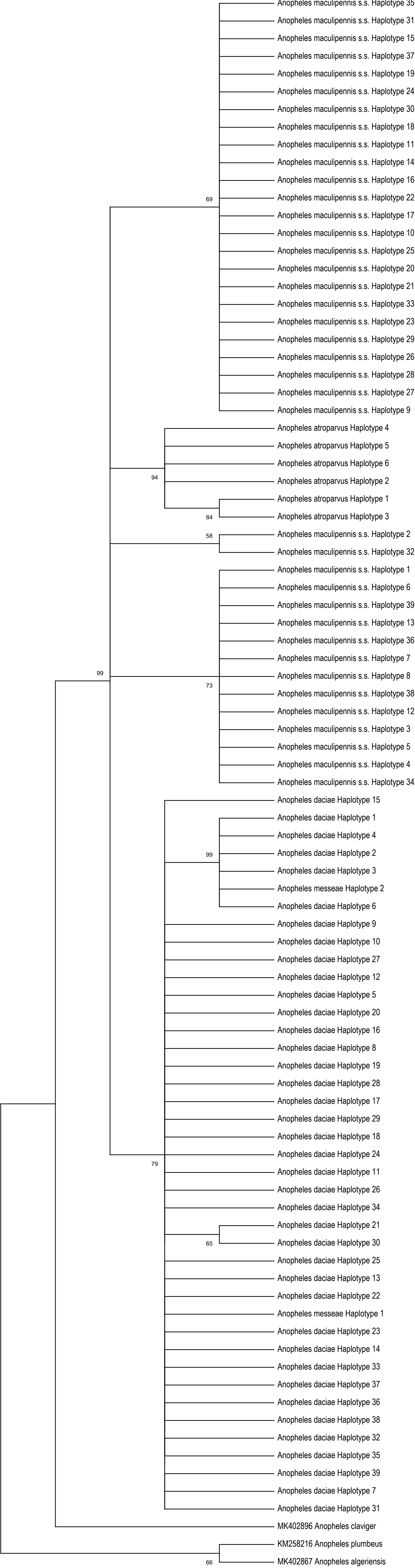

Supplement: Supplementary file 4 — Fig. S2. Condensed COI haplotype ML‐tree of four members of Anopheles maculipennis s.l. in Belgium (Tamura‐Nei model), with An. plumbeus, An. claviger and An. algeriensis as outgroup. Numbers at nodes are bootstrap support values >50%. [file MVE-35-442-s003.pdf]
