# Supplementary material for: DNA identification of species of the Anopheles maculipennis complex and first record of An. daciae in Belgium
Source: Med Vet Entomol. 2021 May 5;35(3):442–50. doi: 10.1111/mve.12519 (PMC8453948; doi:10.1111/mve.12519)
Supplement: Supplementary file 5 — Table S1. Sampling locations (Fig. 1), Corine Land Cover Class (class with highest percentage in a 2.5 km buffer zone around the location (group levels based on five classes, Table S2)) and DNA‐based identification results of specimens collected in Belgium from 2007 until 2019 with indication of the life stage at collection (A, number of adult specimens; L, number of larvae), the adult trap type which collected the specimens and the type of sampled breeding site positive to Anopheles maculipennis s.l. larvae. When multiple coordinates are reported for one location in the table, the middle point between the coordinates was used to position the pie chart on Fig. 1. MMT, mosquito magnet trap; BG, BG‐sentinel trap; GT, gravid trap. [file MVE-35-442-s006.docx]

| Map code | Location | Latitude | Longitude | Main Corine Land Cover Class (percentage) | *An. maculipennis* s.s. | *An. daciae* sp. inq. | *An. messeae* | *An. atroparvus* | TOTAL |
| --- | --- | --- | --- | --- | --- | --- | --- | --- | --- |
| 1.a | Natoye 1 | 50.339201 | 5.045740 | Agricultural areas (77 %) | 6L (tyre, plastic sheet) | 0 | 0 | 0 | 6 |
|  | Natoye 2 | 50.335863 | 5.071636 | Agricultural areas (75 %) | 1A (MMT) | 0 | 0 | 0 | 1 |
| 1.b | Assesse | 50.357190 | 5.078226 | Agricultural areas (80 %) | 1L (metal container) | 1L (metal container) | 0 | 0 | 2 |
| 2. | Frameries | 50.412026 | 3.924913 | Agricultural areas (65 %) | 2A (MMT, BG), 6L (road drain, metal container) | 2A (MMT) | 0 | 0 | 10 |
| 3. | Rocherath | 50.428931 | 6.283842 | Agricultural areas (81 %) | 4L (tyre) | 0 | 0 | 0 | 4 |
| 4. | Marchin | 50.493840 | 5.219160 | Agricultural areas (41 %) | 4A (MMT) | 1A (MMT) | 0 | 0 | 5 |
| 5. | Villers-Le-Bouillet | 50.585328 | 5.259748 | Agricultural areas (77 %) | 1L (tyre) | 0 | 0 | 0 | 1 |
| 6. | Eupen | 50.632399 | 6.073232 | Forest and seminatural areas (61 %) | 24A (MMT), 1L (plastic container) | 0 | 0 | 0 | 25 |
| 7. | Grâce-Hollogne | 50.637785 | 5.431387 | Urban (50 %) and agricultural (50 %) areas | 1A (MMT) | 0 | 1A (MMT) | 0 | 2 |
| 8. | Rekkem | 50.767393 | 3.172912 | Agricultural areas (57 %) | 1L (ditch) | 0 | 0 | 0 | 1 |
| 9. | Muizen | 51.000630 | 4.517250 | Urban areas (46 %) | 0 | 1A (MMT) | 0 | 0 | 1 |
| 10a. | Maasmechelen | 50.995255 | 5.621248 | Forest and seminatural areas (67 %) | 2A (MMT, GT) | 4A (MMT) | 0 | 0 | 6 |
| 10.b. | Dilsen-Stokkem 1 | 51.015500 | 5.757890 | Agricultural areas (71 %) | 0 | 1A (MMT) | 0 | 0 | 1 |
|  | Dilsen-Stokkem 2 | 51.014718 | 5.689910 | Forest and seminatural areas (56 %) | 10A (MMT), 7L (metal and plastic containers, tyre) | 14A (MMT) | 0 | 0 | 31 |
|  | Dilsen-Stokkem 3 | 51.016278 | 5.695222 | Forest and seminatural areas (50 %) | 15A (MMT) | 29A (MMT) | 0 | 0 | 44 |
|  | Dilsen-Stokkem 4 | 51.002758 | 5.632642 | Forest and seminatural areas (64 %) | 1L (stone container) | 0 | 0 | 0 | 1 |
|  | Dilsen-Stokkem 5 | 51.038520 | 5.669410 | Forest and seminatural areas (53 %) | 2A (MMT) | 0 | 0 | 0 | 2 |
| 11. | Grembergen | 51.042310 | 4.065930 | Agricultural areas (76 %) | 0 | 0 | 1A (MMT) | 0 | 1 |
| 12. | Houtvenne | 51.071230 | 4.799080 | Agricultural areas (53 %) | 1A (MMT) | 0 | 0 | 0 | 1 |
| 13. | Lochristi | 51.112279 | 3.836114 | Agricultural areas (74 %) | 1A (MMT), 1L (plastic container) | 0 | 0 | 0 | 2 |
| 14.a. | Kallo 1 | 51.251563 | 4.217950 | Urban areas (44 %) | 5A (BG) | 7A (BG) | 0 | 5A (BG, MMT) | 17 |
|  | Kallo 2 | 51.277853 | 4.278831 | Urban areas (68 %) | 0 | 1L (pond) | 0 | 0 | 1 |
| 14.b. | Vrasene | 51.213511 | 4.193505 | Agricultural areas (86 %) | 2A (MMT, BG) | 1A (MMT) | 0 | 1A (MMT) | 4 |
| 15. | Antwerp | 51.216938 | 4.423997 | Urban areas (96 %) | 5A (MMT) | 0 | 0 | 0 | 5 |
| 16. | Charleroi | 50.462537 | 4.471792 | Urban areas (80 %) | 1L (ditch) | 0 | 0 | 0 | 1 |
| **TOTAL** | | | | | **105** | **62** | **2** | **6** | **175** |
