# Supplementary material for: DNA identification of species of the Anopheles maculipennis complex and first record of An. daciae in Belgium
Source: Med Vet Entomol. 2021 May 5;35(3):442–50. doi: 10.1111/mve.12519 (PMC8453948; doi:10.1111/mve.12519)
Supplement: Supplementary file 6 — Table S2. Corine Land Cover Classes in a 2.5 km buffer zone around the locations with Anopheles maculipennis s.l. collections, with indication of the highest percentage (bold). [file MVE-35-442-s002.docx]

| Location | Urban areas | Agricultural areas | Forest and seminatural areas | Wetlands | Waterbodies |
| --- | --- | --- | --- | --- | --- |
| Antwerp | **0.956** |  |  |  | 0.044 |
| Assesse | 0.071 | **0.803** | 0.125 |  |  |
| Charleroi | **0.801** | 0.195 | 0.004 |  |  |
| Dilsen-Stokkem 1 | 0.188 | **0.705** |  |  | 0.108 |
| Dilsen-Stokkem 2 | 0.291 | 0.093 | **0.555** |  | 0.061 |
| Dilsen-Stokkem 3 | 0.323 | 0.131 | **0.500** |  | 0.045 |
| Dilsen-Stokkem 4 | 0.236 | 0.126 | **0.635** |  | 0.004 |
| Dilsen-Stokkem 5 | 0.084 | 0.382 | **0.534** |  |  |
| Eupen | 0.162 | 0.198 | **0.610** |  | 0.031 |
| Frameries | 0.320 | **0.647** | 0.033 |  |  |
| Grembergen | 0.221 | **0.763** |  |  | 0.015 |
| Grâce-Hollogne | **0.503** | 0.497 |  |  |  |
| Houtvenne | 0.369 | **0.528** | 0.103 |  |  |
| Kallo 1 | **0.437** | 0.325 | 0.132 | 0.007 | 0.098 |
| Kallo 2 | **0.676** | 0.027 | 0.188 |  | 0.109 |
| Lochristi | 0.255 | **0.737** |  |  | 0.009 |
| Maasmechelen | 0.241 | 0.094 | **0.665** |  |  |
| Marchin | 0.259 | **0.408** | 0.333 |  |  |
| Muizen | **0.458** | 0.440 | 0.050 |  | 0.051 |
| Natoye 1 | 0.112 | **0.773** | 0.115 |  |  |
| Natoye 2 | 0.082 | **0.749** | 0.169 |  |  |
| Rekkem | 0.428 | **0.572** |  |  |  |
| Rocherath | 0.108 | **0.813** | 0.079 |  | 0.001 |
| Villers-Le-Bouillet | 0.203 | **0.766** | 0.031 |  |  |
| Vrasene | 0.142 | **0.858** |  |  |  |
