# Supplementary material for: DNA identification of species of the Anopheles maculipennis complex and first record of An. daciae in Belgium
Source: Med Vet Entomol. 2021 May 5;35(3):442–50. doi: 10.1111/mve.12519 (PMC8453948; doi:10.1111/mve.12519)
Supplement: Supplementary file 7 — Table S3. Pairwise F ST estimates between species of the An. maculipennis complex based on COI, calculated using Arlequin v3.5. Significant values after standard Bonferroni correction marked by an asterisk (P < 0.0005). [file MVE-35-442-s005.docx]

| *F_ST_* | *An. atroparvus* | *An. daciae* sp. inq. | *An. maculipennis* s.s. | *An. messeae* |
| --- | --- | --- | --- | --- |
| *An. atroparvus* | 0 |  |  |  |
| *An. daciae* sp. inq. | 0.697* | 0 |  |  |
| *An. maculipennis* s.s. | 0.608* | 0.583* | 0 |  |
| *An. messeae* | 0.671 | 0.028 | 0.540 | 0 |
